# Revisiting the Relationship between Individual Differences in Analytic Thinking and Religious Belief: Evidence That Measurement Order Moderates Their Inverse Correlation

**DOI:** 10.1371/journal.pone.0138922

**Published:** 2015-09-24

**Authors:** Anna J. Finley, David Tang, Brandon J. Schmeichel

**Affiliations:** Texas A&M University, College Station, TX, United States of America; University of Amsterdam, NETHERLANDS

## Abstract

Prior research has found that persons who favor more analytic modes of thought are less religious. We propose that individual differences in analytic thought are associated with reduced religious beliefs particularly when analytic thought is measured (hence, primed) first. The current study provides a direct replication of prior evidence that individual differences in analytic thinking are negatively related to religious beliefs when analytic thought is measured before religious beliefs. When religious belief is measured before analytic thinking, however, the negative relationship is reduced to non-significance, suggesting that the link between analytic thought and religious belief is more tenuous than previously reported. The current study suggests that whereas inducing analytic processing may reduce religious belief, more analytic thinkers are not necessarily less religious. The potential for measurement order to inflate the inverse correlation between analytic thinking and religious beliefs deserves additional consideration.

## Introduction

Dual process models of cognition suggest that human thought can be categorized into analytic, rational processes and intuitive, experiential processes [1, 2]. The intuitive, experiential System 1 is theorized to operate automatically, with little to no conscious thought. System 1 contains processes common to other animals, including emotional and experiential processes [2]. By contrast, the more logical System 2 is thought to underlie distinctly human processes such as abstract reasoning and deliberate, conscious thought [2].

Research has suggested that intuitive thought processes underlie religious beliefs [3, 4, 5, 6]. Beliefs about mind-body dualism, agency, and morality have all been linked to intuitive processes [5] and are also important for religious beliefs [6]. Furthermore, young children rely mainly on intuitive systems [7] and also show a propensity towards body-soul dualism and sensitivity to agency [4]. Thus, the intuitive System 1 appears to undergird at least some formative aspects of religious beliefs.

More direct evidence for the relationship between dual process models and religious beliefs was reported by Gervais and Norenzayan (Study 1), who found that individual differences in intuitive versus analytic processing correlate with religious beliefs, such that more analytic thinkers endorse less religious beliefs [8]. Other researchers have found similar negative correlations between analytic thinking and religious beliefs [9, 10, 11, 12]. Taken together, these findings suggest that persons who favor deliberate, logical thought are less swayed by religious faith.

We propose that the relationship between individual differences in analytic thought and religious beliefs is not as strong as previous evidence has suggested. More precisely, we propose that the negative correlation between analytic thought and religious beliefs is strongest when individuals have engaged the analytic system prior to reporting their religious beliefs. Thus, the measurement order used in Gervais and Norenzayan (Study 1) may have inflated the strength of the relationship between analytic processing and religious beliefs.

Specifically, Gervais and Norenzayan (Study 1) had participants complete the cognitive reflection test (CRT) [13] before reporting their religious beliefs [8]. The CRT consists of three word problems that require analytic processing to solve correctly. Although ambiguity exists regarding the extent to which analytical thought processes are engaged when participants give incorrect answers on the CRT [14, 15], it is clear that arriving at correct answers on the CRT requires analytic thought. Therefore, scores on the CRT may have negatively predicted religious beliefs in the study by Gervais and Norenzayan because correctly answering CRT questions primed analytic processing prior to the measure of religious beliefs. This idea is bolstered by the follow-up studies reported by Gervais and Norenzayan, in which experimental inductions to increase analytic processing (e.g., experiencing cognitive disfluency as in Study 5) reduced religious beliefs [8].

Indeed, several prior experiments have used the CRT to activate an analytic mindset. For example, research by Paxton, Ungar, and Greene found that completing the CRT before making moral judgments led to a positive correlation between analytic thinking and utilitarian (i.e., less emotional) moral reasoning, whereas the correlation disappeared if the moral reasoning task was completed first [16]. These results imply that the observed differences in moral reasoning were due to the temporary activation of an analytic mindset upon completing the CRT first, rather than stable individual differences in analytic thinking.

By having participants complete the CRT prior to reporting religious beliefs, Gervais and Norenzayan likely activated an analytic mindset in participants who answered analytically (i.e., correctly), temporarily suppressing religious beliefs [8]. As a result, the strength of the relationship between individual differences in analytic thinking and religious beliefs may have been inflated by first activating the analytic system, rather than representing a pure (i.e., confound-free) relationship between individual differences in analytic thinking and religious beliefs.

The current study had two main goals. First, we sought a direct replication of the finding that individual differences in analytic thinking are negatively related to religious beliefs when the two constructs are measured in the typical order (i.e., analytic thinking assessed first) [8]. Second, we tested the hypothesis that the relationship between religious beliefs and analytic thought is reduced when religious beliefs are measured first. We registered these hypotheses on Open Science Framework (https://osf.io/v34gw/) prior to data analysis. We found support for both predictions, which suggests that the relationship between individual differences in analytic thinking and religious beliefs is influenced by measurement order. Furthermore, in light of prior evidence linking the need for cognition (NFC) and religiosity [11], we also included a measure of need for cognition (NFC) at the end of the study to allow for exploratory analyses probing the relationship between NFC and religious beliefs; these analyses yielded additional support for the measurement order hypothesis.

## Method

### Ethics statement

The study was approved by the Institutional Review Board (IRB) at Texas A&M University. A waiver of written informed consent was approved by the IRB because the study involved no more than minimal risk to participants. Participants read an information sheet presented before they could access the study. If participants consented to participate, they clicked a button and the study began. Participants’ responses thus reflect documentation of consent.

### Participants

Gervais and Norenzayan (Study 1) sampled 179 students [8]. We aimed to sample at least as many participants for each of the two conditions in the current study. To account for incomplete responses we oversampled, and 454 undergraduate students enrolled in an introductory psychology class accessed the study online. Twelve students did not complete the study and 32 failed the attention check (see below). The remaining 410 participants (318 female, 275 Caucasian, *M* = 18.55 years, *SD* = 1.02 years) were randomly assigned between the analytic first (*n* = 205) or religious beliefs first (*n* = 205) conditions.

### Materials and Procedures

#### Analytic task

Participants completed the cognitive reflection test (CRT), which consists of three logic puzzles that can be answered analytically or intuitively [13]. For example, the question, “A bat and a baseball cost $1.10 in total. The bat costs $1.00 more than the ball. How much does the ball cost?” brings to mind the intuitive answer of $0.10. However, after further examination, it is apparent that the correct answer is $0.05. For all three questions on the CRT, the intuitive responses are incorrect. The number of correct answers formed the measure of analytic thinking (*M* = 0.74, *SD* = 0.92).

#### Religious beliefs measure

As in Gervais and Norenzayan [8], participants completed measures of intrinsic religiosity [17] (e.g. “My faith involves all of my life”; *M* = 4.70, *SD* = 1.58, α = .94), intuitive religious belief [8] (e.g. “I just don’t understand religion” reversed coded; *M* = 5.77, *SD* = 1.32, α = .87), and belief in supernatural agents [8] (e.g. “God exists”; *M* = 6.05, *SD* = 1.53, α = .93), all anchored from 1 (*Strongly Disagree*) to 7 (*Strongly Agree*). The three scales were highly interrelated (*r*s between .69 and .85, *p*s < .001). We calculated a composite score combining all three scales (*M* = 5.22, *SD* = 1.41, α = .96).

#### Final questionnaires

Last, participants reported demographic information and completed the need for cognition (NFC) scale [18] (e.g. “I find satisfaction in deliberating hard and for long hours”; *M* = 3.26, *SD* = 0.65, α = .88), the brief self-control scale [19], and encountered an attention check requiring a click on a circle instead of a Likert rating [20]. Participants who failed the attention check were excluded from all analyses. We present results for the NFC scale below, but the other questionnaire (i.e., the brief self-control scale) was included for exploratory purposes will not be discussed further.

#### Statistical programs

All statistics reported except for the bootstrapped analysis were calculated using SPSS Version 22.0 [21]. The bootstrapped analysis was performed in R [22].

## Results

### Preliminary analyses

We first performed a chi-square analysis to ensure that the distribution of analytic answers on the CRT did not differ between the analytic first and religious beliefs first conditions. The chi-square test was non-significant, χ^2^ (3) = 3.28, *p* = .35, indicating that the proportion of analytic answers on the CRT did not differ by condition. Next, we examined mean responses on all variables of interest to determine if they differed by condition. Please refer to Table 1. As expected, there were no significant mean differences by condition on the number of analytic answers on the CRT, *t* (408) = -0.69, *p* = .488; NFC, *t* (408) = 0.46, *p* = .646; intrinsic religiosity, *t* (408) = 0.56, *p* = .576; intuitive religious beliefs, *t* (408) = 0.36, *p* = .721; belief in supernatural agents, *t* (408) = -0.84, *p* = .401; or the composite religiosity scale, *t* (408) = -0.40, *p* = .689.

**Table 1:** Composite Religiosity Scores as a Function of Condition and Number of Analytic Answers on the CRT.

	Number of Analytic Answers on CRT				
Condition	0	1	2	3	Total
Analytic First	*n* = 110 5.36 (0.12)	*n* = 52 5.30 (0.18)	*n* = 35 4.76 (0.28)	*n* = 8 4.14 (0.67)	*n* = 205 5.19 (0.10)
Religious Beliefs First	*n* = 107 5.27 (0.13)	*n* = 53 5.09 (0.20)	*n* = 29 5.50 (0.30)	*n* = 16 5.22 (0.36)	*n* = 205 5.25 (0.10)
Total	*n* = 217 5.31 (0.09)	*n* = 10 5.19 (0.13)	*n* = 64 5.09 (0.21)	*n* = 24 4.86 (0.33)	*n* = 410

Number of participants answering 0, 1, 2, or 3 CRT items correctly in each condition, followed by their average religiosity composite score (with standard errors in parenthesis).

### Main analyses

Our primary interest was the relationship between analytic thinking (i.e., CRT scores) and religious beliefs. We examined this relationship as a function of experimental condition. In the analytic first condition, we found significant negative correlations between analytic thinking as measured by the CRT and religious beliefs: *r*_*Religiosity*_ (205) = -.17, *p* = .018; *r*_*Intuitive*_ (205) = -.23, *p* = .001; *r*_*Agents*_ (205) = -.19, *p* = .006. These results directly replicated Gervais and Norenzayan (Study 1) [8].

When participants completed the religious beliefs measures first, however, scores on the CRT were no longer related to religious beliefs: *r*_*Religiosity*_ (205) = .04, *p* = .603; *r*_*Intuitive*_ (205) = .004, *p* = .95; *r*_*Agents*_ (205) = -.03, *p* = .725. Using Fisher’s *r*-to-*z* transformation we found that the correlations differed significantly between the conditions for two of the religiosity measures, *z*_*Religiosity*_ = 2.05, *p* = .040; *z*_*Intuitive*_ = 2.34, *p* = .019, and were not significantly different for the third, *z*_*Agent*_ = 1.71, *p* = .087.

To further examine the relationship between CRT scores and religiosity, we regressed the religiosity composite score on task order (analytic first or religious beliefs first), analytic thinking score, and their interaction. A bootstrapped regression with 9,999 iterations showed minimal estimate bias (*B*_*Bias*_ < 0.002) [23], so only the standard regression estimates are reported. As shown in Table 2, we found a significant effect for analytic thinking (i.e., scores on the CRT), and a significant interaction between task order and analytic thinking.

**Table 2:** Regression Analysis for Analytic Thinking and Religiosity (n = 410).

	Religiosity Composite Score				
Variable	*B*	SE(*B*)	β	*t*	*p*
Analytic Thinking Score	-0.311	0.110	-.205	-2.81	.005
Task Order	-0.184	0.177	-.066	-1.04	.300
Task Order X Analytic Thinking	0.335	0.150	.186	2.23	.026
	*R* = .140				

**Fig 1:**
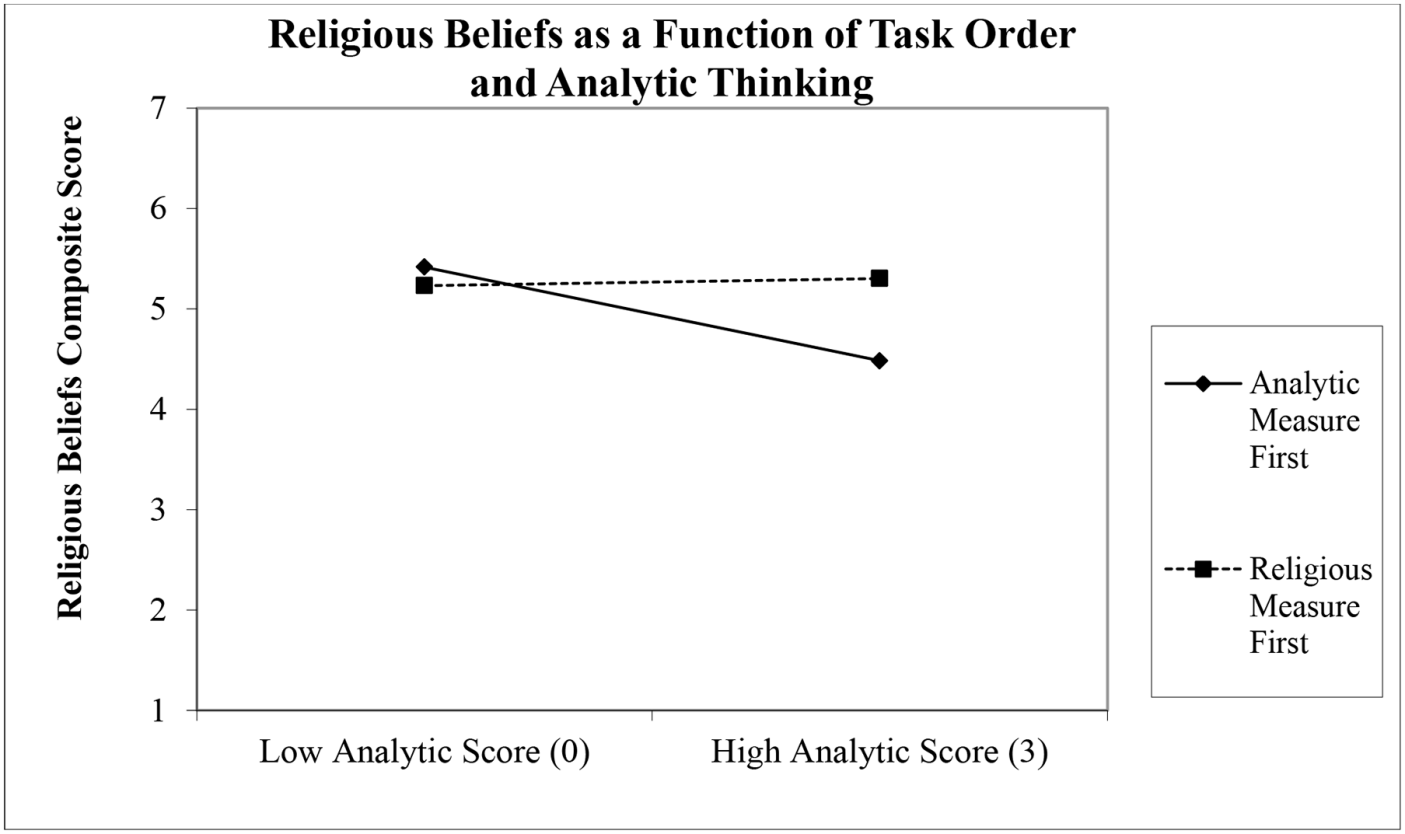
Religious Beliefs as Function of Task Order and Analytic Thinking. Religious beliefs composite score as a function of task order and analytic thinking (CRT score). The solid line is the analytic first condition, whereas the dashed line is the religious beliefs first condition.

To probe the two-way interaction we calculated simple slopes at 1 *SD* above and below the mean in analytic thinking. In the analytic first condition the relationship between analytic thinking and religious beliefs was significant and negative, *b* = -0.31, *t* (406) = 2.81, *p* = .005, whereas in the religious beliefs first condition the same relationship was non-significant, *b* = 0.02, *t* (406) = 0.23, *p* = .815. See Fig 1.

### Secondary analyses

We further examined the relationship between analytic thinking and religious belief by operationalizing analytic thinking as scores on the need for cognition (NFC) scale. Presumably, individuals higher in NFC engage in more analytic thinking when they complete the CRT than do individuals lower in NFC. If that is the case, then NFC should be negatively correlated with religious belief, particularly in the analytic first condition.

Indeed, when participants completed the analytic thinking task first we found significant negative correlations between NFC and intrinsic religiosity, *r* (205) = -.18, *p* = .011, and NFC and belief in supernatural agents, *r* (205) = -.17, *p* = .017, respectively, and a non-significant negative correlation between NFC and intuitive religious beliefs, *r* (205) = -.12, *p* = .080. These patterns mirror findings reported by Pennycook et al. (Study 1) [11].

When participants completed the religious beliefs measures first, however, NFC was no longer correlated with any of the religious beliefs measures: intrinsic religiosity *r* (205) = .06, *p* = .394; intuitive religious beliefs *r* (205) = .06, *p* = .384; belief in supernatural agents *r* (205) = .03, *p* = .647. The correlations differed between the conditions for intrinsic religiosity *z* = 2.43, *p* = .015, and belief in supernatural agents *z* = 2.03, *p* = .042, and were non-significant for intuitive religious beliefs *z* = 1.82, *p* = .069. Hence, the results are virtually the same regardless of whether CRT performance or NFC is used as the measure of analytic thinking.

Because CRT scores and NFC were positively correlated in both experimental conditions (analytic first *r* = .23 *p* = .001; religious beliefs first *r* = .337 *p* < .001), we repeated the regression of the religious belief composite on CRT scores, this time controlling for NFC scores. As shown in Table 3, we found a significant effect of CRT scores and a significant task order by CRT score interaction. As before, we conducted simple slopes at one standard deviation above and below the mean for analytic thinking and found a significant negative relationship between religious beliefs and analytic thinking when analytic thinking was measured first, *b* = -0.296, *t* (406) = 2.64, *p* = .009, but no significant relationship between them when religious beliefs were measured first, *b* = 0.103, *t* (406) = 0.986, *p* = .325.

**Table 3:** Regression Analyses for Analytic Thinking and Religiosity, Controlling for Need for Cognition (n = 410).

	Religiosity Composite Score				
Variable	*B*	SE(*B*)	β	*t*	*p*
Need for Cognition	-0.086	0.112	-.040	0.78	.439
Analytic Thinking Score	-0.296	0.112	-.195	2.64	.009
Task Order	-0.191	0.178	-.068	1.07	.284
Task Order X Analytic Thinking	0.339	0.150	.189	2.26	.025
	*R* = .145				

## Discussion

We replicated the negative relationship between analytic thinking and religious beliefs as reported by Gervais and Norenzayan (Study 1) [8], but only when analytic thinking was measured first. Reversing the measurement order reduced the relationship to non-significance. Furthermore, the pattern of results was the same regardless of whether individual differences in analytic thinking were operationalized as scores on the CRT or scores on the NFC. Thus, analytic thinking appears to relate to religious beliefs primarily when the analytic system is primed before assessing religious beliefs. These results suggest that activating analytic thought changes the relationship between analytic thinking and religious beliefs.

We found a significant change in the relationship between analytic thinking and religiosity depending on which measure came first. As can be seen in the registered hypotheses on the Open Science Foundation website (https://osf.io/sdnhv/?view_only=febcb81890264988a19942417be5221b), we did not predict nor did we find an effect of measurement order on mean levels of religiosity or analytic thinking. The implication is that completing an analytic thinking task reduces religious beliefs only among more analytic thinkers. A majority of the participants were decidedly intuitive thinkers (i.e., they answered all the CRT questions incorrectly). Hence we did not expect all participants to show changes in religious beliefs after completing the analytic thinking measure because most participants do not engage analytic thought on the CRT. It is thus not surprising that mean-level religiosity ratings did not differ between the task order conditions.

### Cognitive Reflection Test and analytic thinking

There is some debate in the literature regarding the usefulness of the CRT as a measure of analytic thinking. Specifically, biases in thinking may occur at a variety of processing stages (e.g., failure to draw upon relevant prior knowledge, failure to inhibit alternate strategies), which may have different cognitive ramifications despite yielding the same behavioral outcome [14]. Furthermore, related research using syllogisms and other logic problems to measure analytic thinking cautions against using normative answers to measure analytic thinking, as analytic thought processes may also be activated for incorrect solutions [24]. Nonetheless, the CRT has objectively correct answers that require analytic processing to achieve. It is thus reasonable to assume that, on average, individuals who answer CRT questions correctly engage in more analytic processing relative to those who answer intuitively or provide incorrect answers.

### The relationship between analytic thinking and religiosity

The current investigation was inspired by the studies reported by Gervais and Norenzayan [8], but other studies are also relevant to the current findings. For instance, Pennycook and colleagues reported negative correlations between religious beliefs and analytic thinking as measured by the CRT [10], as did Gervais in two recent studies [25]; however, like Gervais and Norenzayan, these researchers measured analytic thinking with the CRT prior to measuring religious beliefs. Based on the results from the current experiment, we propose that such findings partly reflect the effect of activating the analytic system among more analytic thinkers and do not reflect a pure or confound-free assessment of the relationship between individual differences in analytic thought and religious beliefs.

Some studies have found inverse correlations between analytic thinking and religious beliefs when religious beliefs were measured first. Shenhav, Rand, and Greene (Study 1) found an inverse relationship between the number of analytic responses on the CRT and belief in God despite measuring religious beliefs before the CRT [9]. Research by Pennycook and colleagues found a negative relationship between religiosity and various measures of analytic thinking when religiosity was measured in a prescreening session and analytic thinking was measured at a later point in time (Study 2 and 3); they also found a negative relationship between NFC and religiosity (Study 1) [11]. These results appear to conflict with the current study, which found a negative correlation between analytic responses and religious belief only when participants completed the CRT before reporting their religious beliefs.

Key methodological differences may explain the seemingly different patterns of results. Specifically, Shenhav et al. embedded their key measures among a larger survey that included measures of hypothetical monetary discounting, political attitudes, and responses to different political issues [9]. Because these additional measures took place prior to or between the measures of interest, and because the current results suggest that the link between analytic thinking and religious beliefs is sensitive to measurement order, the different results may stem from the differences in measurements and measurement order. It is plausible, for example, that completing the delay discounting measure induced an analytical mindset prior to the measure of belief in God in Shenhav et al., which may have inflated the relationship they observed between religious beliefs and analytic thinking. Indeed, delayed discounting rates have been associated with increased activity in the prefrontal cortex [26] and with working memory [27], both of which are commonly associated with analytic modes of thought.

Similarly, in Pennycook et al. (Study 1), prior to measuring religious beliefs, [11], participants completed 18 base-rate problems by choosing which of two possible outcomes was more likely. Six of the base-rate problems were specifically designed to be incongruent (i.e., they contained stereotypes that conflicted with base-rate information defined in the problem). Response incongruence has been shown to activate the anterior cingulate cortex (ACC), an area in the brain related to response conflict and performance on executive functioning tasks requiring conflict monitoring [28]. As such, it is possible that the base-rate problems activated the analytic system and thus enhanced the correlation between analytic thinking (as assessed by the NFC scale) and religiosity. Additionally, in the other study reported by Pennycook and colleagues, it is unclear if other measures (e.g., measures that may have induced analytic thought) were administered in the prescreening session prior to the religiosity measure (Study 2) [11]. Given that the results of the current study suggest that responding to only three questions in an analytic manner prior to reporting religious beliefs was sufficient to influence the relationship between analytic thinking and religious beliefs, it is possible that unreported measures in the prescreening session of the studies in Pennycook et al. were sufficient to activate an analytic mindset before religious beliefs were assessed. Without further information, it is difficult to draw clear conclusions regarding the implications of the current findings for the results reported by Pennycook et al. [11].

The current study, unlike the previous studies, measured religious beliefs and analytic thought with no separation or other measures included between them, thereby providing a clean assessment of the two constructs free from any possible interfering effects of other tasks and measures. Given the finding that the relationship between analytic thinking and religiosity was susceptible to order effects, it is clear that future research is needed to further explore the extent to which the relationship between the two constructs is driven by stable individual differences versus situational or contextual priming of more analytic modes of thought. Our findings imply that individual differences play a smaller role than prior research would suggest and highlight the importance of situation or contextual priming.

### Cognitive Reflection Test and order effects

One additional implication of the current research is that studies using the CRT as an individual difference measure of analytic versus intuitive thinking should be mindful of potential order effects. Completing the CRT appears to influence subsequent responding on unrelated tasks, and perhaps especially so when participants arrive at correct (i.e., analytic) responses. Many studies have used the CRT to measure individual differences in analytic and intuitive thinking but have not counterbalanced measurement order or considered the potential effects of administering the CRT prior to other measures of interest [13, 17, 29]. Future research should examine the extent to which activating more analytic modes of thought influences other measures.

## Conclusion

The results of the current study emphasize the dynamic interrelation between intuitive and analytic processing systems, especially in regards to religious beliefs. We replicated the relationship between analytic thought and religious disbelief, but only when analytic thought was measured first. Assessing religious belief prior to assessing analytic thinking reduced this relationship to non-significance. Future research should examine order effects when probing dual process phenomena, especially when using measures that activate analytic thinking styles, such as the CRT. The current results suggest that the relationship between religiosity and analytic thinking is not as straightforward as prior research suggests. More analytic thinkers are not necessarily less religious, and the relationship hinges on what comes first: thinking analytically or thinking about religion.

## References

[1] Epstein S. Cognitive-Experiential Self-Theory of Personality In: Millon T, Lerner MJ, editors. Handbook of psychology. Hoboken, NJ: John Wiley & Sons, Inc;2003 p. 159–84.

[2] Evans JSBT. In two minds: dual-process accounts of reasoning. Trends Cogn Sci. 2003 10;7(10): 454–9. 14550493 10.1016/j.tics.2003.08.012

[3] Barrett JL. Exploring the natural foundations of religion. Trends Cogn Sci. 2000 1;4(1): 29–34. 10637620 10.1016/s1364-6613(99)01419-9

[4] Bloom P. Religion is natural. Dev Sci. 2007 1;10(1): 147–51. 17181713 10.1111/j.1467-7687.2007.00577.x

[5] Baumard N, Boyer P. Religious beliefs as reflective elaborations on intuitions: A modified dual-process model. Curr Dir Psychol Sci. 2013 8;22(4): 295–300.

[6] Aarnio K, Lindeman M. Religious People and Paranormal Believers. J Individ Differ. 2007 1 1;28(1): 1–9.

[7] Jacobs JE, Klaczynski PA. The development of judgment and decision making during childhood and adolescence. Curr Dir Psychol Sci. 2002 8;11(4): 145–9.

[8] Gervais WM, Norenzayan A. Analytic thinking promotes religious disbelief. Science. 2012 4–27;336(6080): 493–6. 10.1126/science.1215647 22539725

[9] Shenhav A, Rand DG, Greene JD. Divine intuition: Cognitive style influences belief in God. J Exp Psychol Gen. 2012;141(3): 423–8. 10.1037/a0025391 21928924

[10] Pennycook G, Cheyne JA, Seli P, Koehler DJ, Fugelsang JA. Analytic cognitive style predicts religious and paranormal belief. Cognition. 2012 6;123(3): 335–46. 10.1016/j.cognition.2012.03.003 22481051

[11] Pennycook G, Cheyne JA, Barr N, Koehler DJ, Fugelsang JA. Cognitive style and religiosity: The role of conflict detection. Mem Cogn. 2013 6 20;42(1): 1–10. 10.3758/s13421-013-0340-7 23784742

[12] Razmyar S, Reeve CL. Individual differences in religiosity as a function of cognitive ability and cognitive style. Intelligence. 2013 9;41(5): 667–73.

[13] Frederick S. Cognitive reflection and decision making. J Econ Perspect. 2005 10;19(4): 25–42.

[14] De Neys W, Bonnefon J-F. The “whys” and “whens” of individual differences in thinking biases. Trends Cogn Sci. 2013 4;17(4): 172–8. 10.1016/j.tics.2013.02.001 23490722

[15] Toplak ME, West RF, Stanovich KE. The Cognitive Reflection Test as a predictor of performance on heuristics-and-biases tasks. Mem Cogn. 2011 5 4;39(7): 1275–89. 10.3758/s13421-011-0104-1 21541821

[16] Paxton JM, Ungar L, Greene JD. Reflection and reasoning in moral judgment. Cogn Sci. 2012 1;36(1): 163–77. 10.1111/j.1551-6709.2011.01210.x 22049931

[17] Hoge DR. A validated intrinsic religious motivation scale. J Sci Study Relig. 1972 12;11(4): 369–76.

[18] Cacioppo JT, Petty RE. The need for cognition. J Pers Soc Psychol. 1982 1;42(1): 116–131.

[19] Tangney JP, Baumeister RF, Boone AL. High self-control predicts good adjustment, less pathology, better grades, and interpersonal success. J Pers. 2004;72(2): 271–324. 15016066 10.1111/j.0022-3506.2004.00263.x

[20] Oppenheimer DM, Meyvis T, Davidenko N. Instructional manipulation checks: Detecting satisficing to increase statistical power. J Exp Soc Psychol. 2009 7;45(4): 867–72.

[21] IBM Corp. IBM SPSS Statistics for Windows, Version 22.0. Armonk, NY: IBM Corp; 2013.

[22] Fox, J. Package ‘car’: Companion to Applied Regression, Version 20–25. Open-Source: R CRAN; 2015.

[23] Kutner MH, Nachtsheim CJ, Neter J, Li W. Applied linear statistical models. 5th ed New York: McGraw-Hill/Irwin; 2005 p. 458–64.

[24] Thompson VA, Prowse Turner JA, Pennycook G. Intuition, reason, and metacognition. Cogn Psychol. 2011 11;63(3): 107–40. 10.1016/j.cogpsych.2011.06.001 21798215

[25] Gervais W. Override the controversy: Analytic thinking predicts endorsement of evolution. Cognition. 2015 5;142: 312–321. 10.1016/j.cognition.2015.05.011 26072277

[26] Monterosso JR, Luo S. An argument against dual valuation system competition: Cognitive capacities supporting future orientation mediate rather than compete with visceral motivations. J Neurosci Psychol Econ. 2010 5;3(1): 1–14. 21909453 10.1037/a0016827 PMC3169839

[27] Bickel WK, Yi R, Landes RD, Hill PF, Baxter C. Remember the future: Working memory training decreases delay discounting among stimulant addicts. Biol Psychiatry. 2011 2;69(3): 260–5. 10.1016/j.biopsych.2010.08.017 20965498 PMC3015021

[28] Carter CS, van Veen V. Anterior cingulate cortex and conflict detection: An update of theory and data. Cogn Affect Behav Neurosci. 2007 12 1;7(4): 367–79. 18189010 10.3758/cabn.7.4.367

[29] West RF, Meserve RJ, Stanovich KE. Cognitive sophistication does not attenuate the bias blind spot. J Pers Soc Psychol. 2012;103(3): 506–19. 10.1037/a0028857 22663351

